# ‘‘I felt marvellous e-cycling. If I had long hair I would have flicked it”: a qualitative investigation of the factors associated with e-cycling engagement among adults with type 2 diabetes

**DOI:** 10.3389/fspor.2023.1150724

**Published:** 2023-09-29

**Authors:** Jessica E. Bourne, Sam Leary, Clare England, Aidan Searle

**Affiliations:** ^1^ Centre for Exercise, Nutrition and Health Sciences, School of Policy Studies, University of Bristol, Bristol, United Kingdom; ^2^ NIHR Bristol Biomedical Research Centre, University Hospitals Bristol and Weston NHS Foundation Trust and University of Bristol, Bristol, United Kingdom; ^3^ Population Health Sciences, University of Bristol, Bristol, United Kingdom

**Keywords:** e-cycling, perceptions, physical activity, type 2 diabetes, qualitative

## Abstract

**Background:**

Physical activity (PA) is a key component in the management of type 2 diabetes. However, this population have low rates of PA engagement. Electrically assisted cycling has been identified as a means through which to increase PA by incorporating activity into daily life, while overcoming some of the barriers to conventional cycling. The determinants of e-cycling among people living with chronic disease are largely unknown. The aim of this research was to explore the determinants of e-cycling among individuals with type 2 diabetes using the Theoretical Domains Framework (TDF) and the Capability, Opportunity and Motivation for Behaviour change model (COM-B). This information is important for determining the suitability of future e-cycling initiatives and, if appropriate, inform future e-cycling interventions.

**Method:**

One-to-one semi structured interviews were conducted with 16 participants from the e-cycling arm of a pilot randomised controlled trial between September 2019 and April 2020. The TDF was used to develop the interview guide. The framework method of analysis was used, incorporating both deductive and inductive reasoning. A conceptual model of the factors that influence e-cycling in this population was created.

**Results:**

The most commonly reported TDF domains were skills, knowledge, belief about capabilities, belief about consequences and environmental context and resources. Specifically, e-bike training facilitated e-cycling engagement by providing participants with the skills, knowledge, and confidence needed to ride the e-bike and ride on the road. In addition, the enjoyment of e-cycling was a key facilitator to engagement. Participants engaged in e-cycling to improve their health rather than for environmental reasons. Most barriers to e-cycling related to the natural or physical environment.

**Conclusion:**

This study provides insight into the personal, social, and environmental factors associated with e-cycling in this population. The findings of this study can be used to develop a more refined e-cycling intervention targeting the factors identified as influencing e-cycling engagement. In addition, this information will help in the selection of mechanistic outcome measures for evaluation.

## 1. Introduction

Type 2 diabetes is a metabolic disease that negatively impacts an individual's physical and mental health (1–6). By 2040, it is estimated that approximately 642 million individuals worldwide will be diagnosed with diabetes, of which 90% will be type 2 diabetes (7). The cost of ongoing treatment and management of type 2 diabetes and its associated complications places considerable strain on health services (8).

Physical activity (PA) is an important lifestyle factor in the prevention and treatment of type 2 diabetes (9–13). However, individuals with type 2 diabetes are less physically active than those without type 2 diabetes and many fail to meet the recommendation of 150-min of moderate to vigorous physical activity (MVPA) per week (14–16). Interventions aimed at increasing PA in this population often require significant contact time and expertise, thereby limiting their scalability (17). Furthermore, individuals often return to prior PA levels when left to self-manage their activity (17–19). As such, there is a need to develop PA interventions that are less labour intensive and that are effective at promoting behaviour change beyond the intervention period.

Active travel is widely recognised nationally and internationally as a means of increasing PA. As such, encouraging active travel is endorsed by the National Institute of Health and Care Excellence (20) and the World Health Organization (21). Among individuals with type 2 diabetes active commuting is associated with increased PA and lower body mass index (22). Despite widespread endorsement, rates of engagement in active travel in the UK and around the world are low (23–25), especially among individuals living with type 2 diabetes (22). Community-based initiatives can serve to increase cycling behaviour (26–29), however it is rarely maintained over time (30, 31). Furthermore, there are several barriers to conventional cycling that could discourage engagement including physical constraints associated with hilly terrain and poor physical fitness, as well as a lack of time and the distance people have to travel (32). These barriers may be accentuated in individuals with type 2 diabetes given their overall lower levels of PA.

Electrically-assisted bicycles (e-bikes; also known as pedelecs) have been identified as an alternative form of active travel that can positively impact health (33) while overcoming some of the commonly reported barriers to conventional cycling. While e-bikes have many similarities with conventional bicycles, the electrical assistance requires less physical effort and leads to greater riding distance and frequency (34–36). Therefore, it is likely that some determinants of conventional cycling are less relevant to e-cycling. Despite the electrical assistance evidence suggests that e-cycling is performed at at least a moderate intensity and leads to similar or slightly lower physical markers of intensity than conventional cycling (33). However, given that individuals report e-cycling for longer and more frequently than they do a conventional bicycle, e-cycling is often associated with greater weekly energy expenditure than a conventional bicycle (35, 37).

Research conducted with e-bike owners and those who have been provided with an e-bike as part of an intervention reveals that the ability to ride further, faster, on hillier terrain and to ride with friends and family are common facilitators to e-cycling engagement. Conversely, bad weather, poor infrastructure and theft concerns are common barriers to engagement (34, 36, 38–40). To date, few studies have explored the factors associated with e-cycling among people living with chronic disease for whom engagement in active travel is low (22). People living with chronic conditions may experience e-cycling interventions differently and be impacted by different contextual factors than a healthy adult population. Two studies have specifically explored factors associated with e-bike engagement in people living with chronic disease. Boland and colleagues (41) examined the use of adapted e-bikes in three individuals recovering from a stroke. The level of social support, motivation for riding, level of physical impairment all impacted riding. Among individuals with type 2 diabetes, Searle and colleagues (42) reported that e-cycling was perceived as enjoyable and enabled individuals to cycle with friends and family. However, they did not conduct a comprehensive evaluation of the data to explore the determinants of e-bike use, rather the researchers were interested in understanding how e-cycling impacted participants management of their diabetes. E-cycling may offer an alternative to structured lifestyle interventions for individuals with type 2 diabetes, enabling exercise to be completed outside and incorporated into daily life which may lead to sustained physical activity behaviour.

Understanding how participants experience e-cycling, particularly the barriers and facilitators to riding, will enable the development of a conceptual understanding of the factors that are most influential on e-cycling engagement in this population. The use of behaviour-change theory and models can help in this understanding and identify key intervention components required to bring about change. The Medical Research Council guidance for developing complex interventions outlines that researchers need to have a theoretical understanding of the potential processes of change (43). When little is known about the target behaviour in the population of interest qualitative research is useful to develop a theoretical understanding of the behaviour (44). The Behaviour Change Wheel is a practical intervention design tool which transparently guides researchers through intervention design and delivery. At the core of the BCW is the COM-B model which can be used to determine what needs to change for the desired behaviour to occur (45). The COM-B model proposes that human behaviour is a result of the interaction between capability, opportunity and motivation. Specifically, for a behaviour to occur a person must have the psychological and physical capability to perform the behaviour; the physical and social opportunity to engage in it; and they must have the motivation, either conscious or automatic, to engage. COM-B has been found to be an effective model in explaining physical activity behaviours (46).

The Theoretical Domains Framework (TDF) was designed to understand behaviour theoretically by establishing which processes of change should be targeted (47). The TDF consists of 14 theoretical domains that consider the environmental, social, cognitive and affective influences on behaviour (48). The TDF maps directly onto the COM-B components, enabling expansion of each of these components and assisting in identification of the potential determinants of behaviour. Once identified, this information can be used to guide the selection of quantitative measures to examine potential moderating and mediating effects as part of a full-scale evaluation and/or to inform target areas for intervention in future e-cycling initiatives among individuals living with chronic disease. The primary objective of the study was to explore the factors associated with e-cycling engagement among individuals with type 2 diabetes following trialling an e-bike using the TDF and COM-B model, and to develop a conceptual model of the behaviour.

## 2. Methods

### 2.1. Participants and procedures

One-to-one semi-structured interviews were conducted with individuals who were randomised to the intervention arm of a parallel two-arm pilot randomised controlled trial. The trial compared an e-cycling intervention against a standard-care waitlist control in adults with type 2 diabetes (49). Eligibility for the study included having a clinical diagnosis of type 2 diabetes and being between 30 and 70-years of age. Individuals were ineligible if they self-reported engagement in ≥150-min of MVPA per week (50); took exogenous insulin; had a myocardial infarction or stroke in the past six months or had evidence of end-stage renal failure or liver disease; had uncontrolled hypertension; had any other contraindications to exercise; were not cleared to engage in PA by their GP and/or were unable to read and communicate in English. This single centre study was conducted in the city of Bristol, England. Individuals in the intervention arm received e-bike training consisting of two one-to-one training sessions followed by a 12-week e-bike loan in which they were instructed to use the e-bike as they desired (i.e., no riding goals stipulated). During the e-bike loan participants were offered two further training sessions. Twenty individuals were allocated to the intervention arm. Four participants discontinued with the intervention (reasons included: personal situation [*n* = 2], purchased an e-bike [*n* = 1], undisclosed [*n* = 1]). Seventeen individuals were invited to take part in the interviews of which 16 participated. Interviews were conducted by JEB over the telephone between September 2019 and April 2020, within two weeks of finishing the e-bike trial. The interviews were digitally recorded using encrypted recording devices. The recordings were transcribed verbatim by Transcription UK and stored using NVivo data management software (NVivo10, QSR International, 2012). The transcripts were checked against the original recordings to ensure reliability. Interviews ranged between 33mins and 50mins in length. Ethical approval was obtained from the NHS Health Research Authority Southwest/Central Bristol Research Ethics Committee (Ref: 18/SW/0164) and was sponsored by the University of Bristol.

### 2.2. Interview questions

The interview topic guide was developed using the Theoretical Domains Framework (TDF) (48) and based on guidance by Atkins and colleagues (51). The interview guide included at least one question for each theoretical domain to comprehensively consider the possible influences on e-cycling. The interview guide is provided in the Supplementary Material. Follow-up probes or prompts were included to delve more deeply into each domain (51). The order in which questions were asked was flexible to enable flow during the interview.

### 2.3. Qualitative data analysis

Interview data were analysed using the Framework method (52) and guided by Gale and colleagues seven-stage analysis process (53). The Framework method sits within the family of analyses methods known as ‘thematic analysis” (53, 54) and is suited to research that has specific questions and a pre-defined sample (55). Framework analysis does not require adherence to either inductive or deductive analysis approaches and is therefore appropriate in the current theory-based study. In addition, it does not prescribe to a single epistemological or ontological framework thereby providing a degree of flexibility regarding how data analyses is approached (54). Table 1 outlines the steps involved in analysing the data. JEB created the e-cycling intervention as part of her PhD research. She had training in qualitative research methods, developed the interview guide and conducted the interviews. JEB analysed the data with in-depth discussions with AS. In the current study a critical realist ontology and constructionist epistemology was adopted regarding the data.

**Table 1:** The seven stages of the framework method of qualitative analysis and how they were applied to the research question.

Procedure for analysis	Application in the current study
Stage 1. Transcription	All interviews were conducted by JEB and transcribed by Transcription UK. The transcripts were checked against the original recordings to ensure reliability.
Stage 2. Familiarisation	JEB listened to each audio recording and read each transcript. AS read two interview transcripts. The transcripts were selected by JEB to represent diverse experiences of e-cycling.
Stage 3 and 4. Development of analytical framework and coding	Excerpts of the two transcripts were deductively assigned into one or more of the 14 domains reflected in the TDF and directly onto the six components of the COM-B model (content analysis). Excerpts that were deemed important but did not fit into one of the 14-domains were placed in an ‘other’ category. This was done independently by JEB and AS who met to discuss assignment. Following this, inductive coding within each TDF domain took place to identify themes. Codes were noted as either a ‘barrier’ or ‘facilitator’ depending on the context in which the code occurred. These were operationalised as any factor, characteristic, view or belief that either impeded or enabled e-cycling engagement. This was done independently by JEB and AS who met to discuss assignment and an analytical framework was developed. The two researchers independently coded two more transcripts, noting any new codes. The researchers met again to discuss the coding and to revise the initial framework to incorporate new or redefined themes.
Stage 5. Applying the analytical framework (Indexing)	JEB used this framework to code the remaining transcripts using NVivo software. If a new theme was required, this was discussed with AS before adding it to the analytical framework. If a new theme or domain (which did not fit the TDF) was added, previously coded transcripts were checked for relevant data.
Stage 6. Charting data into the framework matrix	After finalising themes, TDF domains and COM-B constructs, a framework matrix was developed. NVivo was used to create matrices that encapsulated data from each domain and sub-domain. Following this, each participants data was described and summarised to develop a chart. This was conducted in Excel and consisted of participants in rows with summaries of domains and sub-domains in columns. AS checked the summaries of the first two transcripts to ensure they captured the essence of the data. Two participants were provided with a copy of their interview transcript and an interpretation of their data. They were asked to review their transcribed data and comment if they felt the interpretations represented or misrepresented their views.
Stage 7. Mapping and interpreting the data	Summative content analysis (56) was applied in which the frequency count of each theme was calculated. TDF domains, and associated COM-B constructs, were judged based on the frequency count of coding for each TDF domain. This frequency coding enabled the identification of the main barriers and facilitators to engaging in e-cycling in this population. The significance and implications of the domains, and how they relate to one another was examined narratively, and a conceptual model of the factors that impact e-cycling in this population was developed.

## 3. Results

### 3.1. Participants characteristics

Of the 16 participants interviewed, there was an equal split between men and women and a mean age of 59.75 years (Standard Deviation = 6.85). Ten participants were working either full or part time. Fourteen participants had some degree of cycling experience prior to the trial. Overall 56.25% of participants completed three or more cycling lessons with the instructors. Fifteen participants had at least one private vehicle at their residence. The distances travelled on the e-bike during the loan period by the 13 participants with available data ranged from 9 km to 1,878 km (Median = 144.4; IQR: 117.0, 307.0), with a median of 22 journeys (IQR: 13, 33) over the loan period based on travel log books. Table 2 provides the demographic characteristics of participants.

**Table 2:** Demographic characteristics.

Variable	*N*
Age, years, mean (SD)	59.75 (6.85)
Sex, %	
Female	8 (50)
Male	8 (50)
Ethnicity, %	
White	15 (93.75)
Asian	1 (6.25)
Employment status, %	
Full-time (35 h or more per week)	7 (43.75)
Part-time	3 (18.75)
Unemployed	1 (6.25)
Retired	5 (31.25)
Previous cycling experience, %	
None	2 (12.5)
A little	9 (56.25)
A lot	5 (31.25)
Number of journeys, median, IQR	22 (13, 33)
Distance ridden, km, %	
0–99	3 (18.75)
100–199	5 (31.25)
200–299	1 (6.25)
300–399	2 (12.5)
400–499	0 (0)
500+	2 (12.5)
Distance not available	3 (18.75)

### 3.2. Summary of the TDF and COM-B model

**Figure 1:**
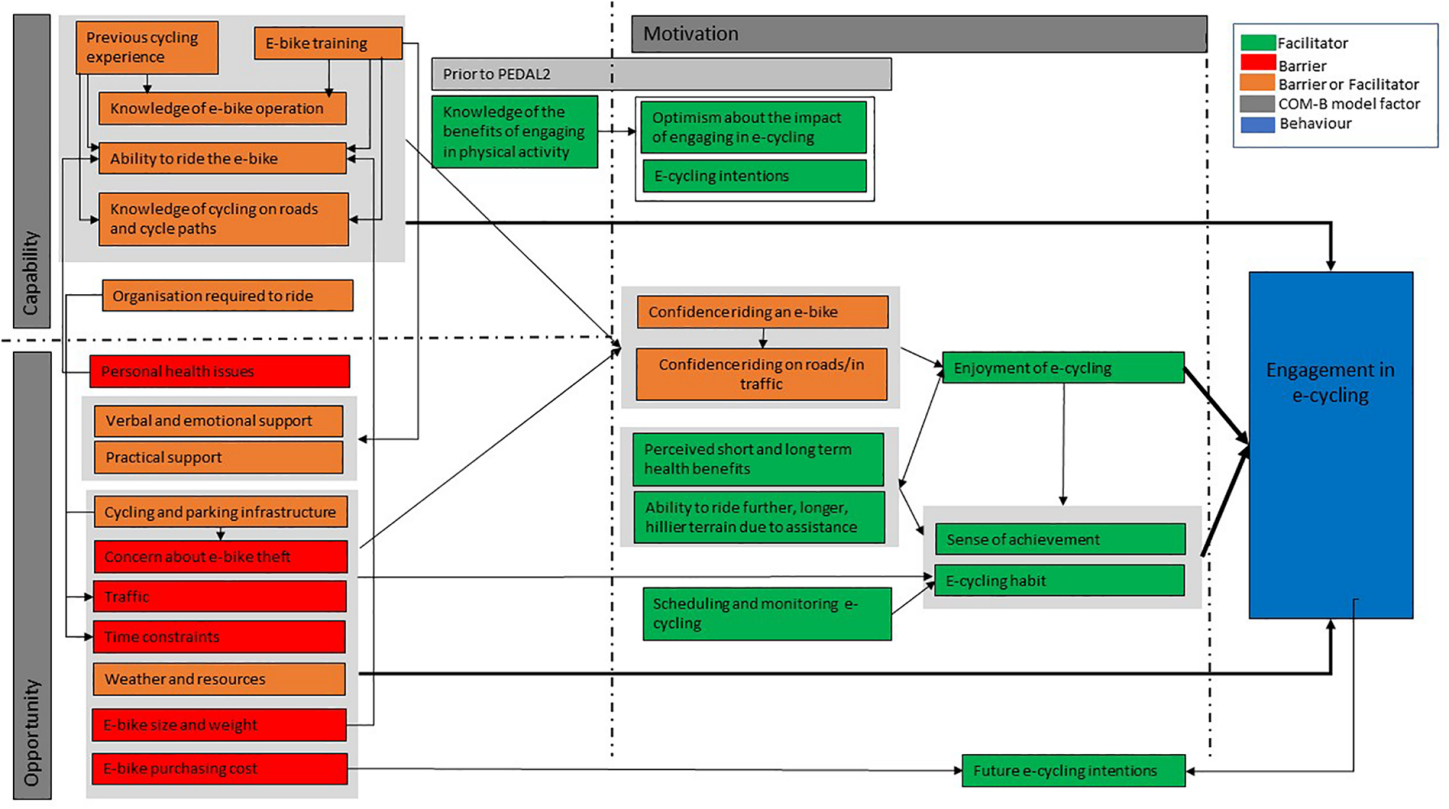
Conceptual model of e-cycling in adults with type 2 diabetes.

The transcripts provided data that represent all the fourteen domains of the TDF and all components of the COM-B model. Table 3 presents a summary of COM-B constructs, TDF domains and frequency counts, themes and example quotes. Whether the theme was identified as a barrier or facilitator to e-cycling engagement is reported. The COM-B constructs, TDF domains and how they relate to one another are summarised narratively and a conceptual model proposed (Figure 1).

The most commonly reported domains were skills, knowledge, beliefs about consequences, belief about capabilities and environmental context/resources, and the least commonly reported domains were optimism, goals and behavioural regulation.

#### 3.2.1. Capability

##### Physical capability: *skills*

Fifteen participants were able to ride the e-bike by the end of the training period. Of these 15, 14 had some degree of previous cycling experience. Participants with more recent cycling or motorcycling experience (two to three years) reported greater ability to ride the e-bike than those with no experience or who had not ridden for a long time (Table 3.1.a.iii). While several participants engaged in frequent cycling at a younger age, and reported confidence riding a bicycle, many were surprised at how different e-cycling was to conventional cycling. This was attributed to the devices themselves, the poor conditions of the roads and the amount of traffic compared to when they used to cycle. As such, some participants felt their previous riding skills did not translate to e-cycling in the present day.

**Table 3:** COM-B constructs, TDF domains, sub-themes and example quotes.

COM-B construct	TDF domain (frequency count)	Sub-themes (frequency count) (Barrier or facilitator)	Example quote
1. Physical capability	a. Skills (107)	**i. Ability to ride and manoeuvre the e-bike** (35) (B, F)	*So at first I was wobbly, even riding round the park I was wobbling. Then, after that, you just got used to it, and it was like just round, and round, and round. It was fine, got on really fine* (P6, no cycling experience); *I found it was different to my ordinary bike in that I was… oh, how do I explain this? I was nearer the handlebars so my legs were going up and down, rather than forwards, if you see what I mean? So, I didn't like* (P14, lots of cycling experience)
		**ii. E-bike training was helpful or insufficient** (40) (B, F)	*Oh, yes. That* [the training] *was really useful actually because I was a bit, you know, “I don't need to do this,” kind of, thing “I've always cycled, I don't need to be shown what to do.” But it was actually quite useful just to do some basics’* (P2, lots of cycling experience); ‘*It was all a bit quick, but I mean- And I thought they should have said something about more training at a later date’* (P15, a little cycling experience)
		**iii. Impact of previous cycling experience** (22) (B, F)	*I had a desire, I had no experience, even in the standby bicycle, or nothing. I think the electronic bike was so heavy and not manageable* (P10); *Well I ride a motorbike anyway, so I'm not unconfident on a road on a bike.* (P1*); In my younger days, I did quite a lot of cycling, so it all seems fairly familiar to me* (P11); *I used to commute but I would say that was say 12 years ago, that was when I lived in London. So, I was a bit surprised how absolutely terrified I was* (P4)
2. Psychological capability	b. Knowledge (141)	**i. Benefits of physical activity** (40) (F)	*Well, every time that you go for a review, they will say, “Losing weight is going to be beneficial. Getting fitter is going to be beneficial,” but it's easier said than done* (P12); *I've only been diabetic for two years. And I think what I’m finding is that it's actually harder than I expected to keep your blood sugars down. It's [exercise] a really positive way of doing that because just to control it through diet is really quite challenging* (P2)
		**ii. Familiarity with cycle routes** (15) (B, F)	*‘Because cycling out there seems to have got better, as in you've got paths to go on, and I found some really interesting paths’* (P3); ‘*I tended to have a particular circuit that was around the XX. Even if it was just forty-five minutes so I had a particular circuit around the XX* (P2)
		**iii. Knowledge of riding on roads** (33) (B, F)	*Training, I feel that was very good at pointing out what you should do at junctions and double checking you're aware of everything and making sure you were looking both ways. Looking and listening and looking again, I think, is very important and they definitely reinforced that.* (P11).
		**iv. Understanding of how to operate the e-bike** (23) (B, F)	*‘When I first started, I was flicking the buttons all the time. But then I realised that you don't have to do that, the bike will kick in by itself. That was absolutely fantastic, I really enjoyed that function. If things got a little bit tricky, it would just kick in and you’d go up a gear and it was brilliant’* (P7); *It took me a good few sessions to sort of get to used to it, the fundamental use of it and how and when to deploy the gears and what have you, and the assistance. But once I got into that pattern, it wasn't too much of a problem* (P16).
	c. Behavioural regulation (9)	**i. Using technology to self-monitor rides** (9) (F)	*‘I go on Strava and you put it on and you get personal records when you do a ride. If I did a trip 20 times, every day I’d have a personal best in there somewhere. And at the hill, there is like 0.1 of a mile up the hill and you get a record, but you knew you did because you were really pushing it. When you get there, you think, “Oh, I actually got the best time.’* (P12)
	d. Memory, attention and decision processes (67)	**i. Remembering to charge e-bike or specific equipment** (18) (B, F)	*‘I wore the helmet all the time, and I went into work and I was coming out and I was like a mile or two miles home and I always wore glasses as well. I could just feel the wind in my hair and I thought, “I haven't got my glasses on. I haven't got my helmet on either.’* (P12, a little cycling experience); ‘*You just got used to getting your bag. Well, the bag was half packed anyway. And I would always take the lock for it. I would always take that with me’* (P3, a little cycling experience): ‘*When I go out anywhere I come home and then I put it straight onto charge, and then it's fully charged’* (P5, a little cycling experience)
		**ii. Decision to engage in e-cycling** (48) (B, F)	*‘I would say that commuting was definitely one reason to get me out on the bike, because it was just very easy to get to work. Where I work is in the middle of town and there isn't any parking allowed, except for special reasons where you have to book it a long time in advance and give a definite reason. There are only four parking spaces for the company, and I think there are probably about 200 employees in the company. All around there the car parking is very, very expensive. So, let's look at it from the other side, the next choices are taking the public transport and the buses are often very full at that commuter time and often go straight past you. So, you can end up not getting the bus. The other option is to walk or drive down to the local train station and, again, the train is very crowded. The final option is to get on the bike, go through a couple of back roads and get on the cycle path, whizz down the cycle path, cross over one major road and then some minor back-roads and there's plenty of bike parking in the cellar of the building and it's secure and monitored with cameras and locked with barriers and automatic gates, so that the bikes are very safe there, so you've got no worry about that. In that respect, it is the best option’* (P11)
3. Physical opportunity	e. Environmental context and resources (265)	**i. Access to infrastructure for riding and parking** (88) (B, F)	*'The only thing storing the bike, I had to put it in the kitchen because I live in a high rise flat, and there's nowhere else to put it’* (P5); *We've got a car park, which is locked. There is an interlock on the back wheel, so I lock that, took the battery off, put the D lock around a fixed clamp, took all the computer off, took the lights off, took the pannier in and left it like that* (P12); ‘*The main way I would have gone into work would have been to go down XX Road. And I don't think there's a cycle lane there and it's very, very busy so… It's put me off. Really’* (P2); ‘*When I’m on early shift, I get up early and I just stick to the roads because I'm not overly confident going on the cycle path at five in the morning in the pitch dark, and coming back at night, I don't really want to be on there at ten or eleven o’ clock either, just all the wispy shadows and people stood behind the bridges and that, it's just a little bit concerning, but I am fine on the roads’* (P12); *That was quite difficult actually* [a particular cycle path] *because it was shared as a footpath. I seemed to be coming across pedestrians every hundred yards’* (P2).
		**ii. E-bike size and design features** (51) (B)	*‘I think the electronic bike was so heavy and not manageable’* (P10); ‘*It was too high, because I didn't feel comfortable not being able to put my feet flat on the floor, so I had to wait for a seat, because the first seat, still my feet didn't touch the floor. Yes, because obviously where I haven't rode a bike, I needed to be able to put my feet on the floor’* (P6); ‘*It was a bit of a pain when you had to… when you locked it up, especially when you were out shopping, to take the battery out, you know, take it off and… because it's quite heavy’* (P1)
		**iii. E-bikes are expensive to buy** (10) (B)	*‘I’d never ridden an e-bike and it was really good. When I had to give the e-bike back I tried to use my normal bike and I couldn't use my normal bike and I was struggling with it. So, I'm going to sell that one and save up for an e-bike, it's really hard trying to get an e-bike because it costs so much money’* (P5)
		**iv. Impact of weather and resources** 51 (B, F)	*‘Because the weather was really bad lately and I couldn't go on the e-bike, so I had to go on the bus to get to places’* (P5); ‘*So the weather, I guess, the only thing that impacted on me later on was in September. Later in September and early October, where it was dark by the time I got home. I didn't enjoy cycling in the dark very much. I did do it sometimes, but not a lot’* (P2); *I did buy waterproofs. How were they? They were alright. They did the job. I was quite pleased with the clothing I bought, it kept the rain out, yes* (P7).
		**v. Personal health issues that impacted riding (**26) (B)	*‘Well the only other barrier I had was this operation I had. The lead up to it I was told not to overdo it and then the operation itself’* (P1); ‘*I had an abscess on my tooth, and then I had to have it come out, that impacted riding’* (P6)
		**vi. Time constraints** (24) (B)	*‘The extra work I was doing at work, I was also tired. I'd always come home, have dinner and a bit of a sleep. So, by the time, say, I felt like going on the bike, it was blinking dark at 4:00pm’* (P13)
		**vii. Traffic concerns** (43) (B)	*Now the roads are getting so busy now with cars and buses, I feel like I'm worried that I'm going to get knocked by a bus or a car* (P5)
		**viii. Concerns about e-bike theft** (21) (B)	***‘****I mean, I have got sisters that live about a mile and a half away, and I didn't even want to go up and see them in it because there was nowhere to put the bike safely. Even with the lock, I wasn't happy about it, you know’*(P15)
4. Social opportunity	f. Social influences (72)	**i. Practical support** (46) (B, F)	*‘No, no, because [partner] used to walk the dogs while I rode around the park. He was always there, so he'd take the dogs, because he’d take the dogs every day anyway, round the park, so he used to do it when I would go’* (P6, female, no cycling experience); ‘*I'm more than happy [to cycle alone], I can stop as and when it suits me’* (P16, male, lots of cycling experience). *‘Well it would be nice to have a family event. We haven't had a family event like that for a long, long time. It would be nice to get out and do things together’* (P7, female, lots of cycling experience); *‘Then in the end we went round Bedminster and I don't think I would have ever cycled around Bedminster without the instructor* (P7).
		**ii. Verbal and emotional support** (29) (B, F)	*‘I think generally positive feedback helps make you, you know, if you are feeling a bit doubtful about whether it was something that was the right thing to do for someone of my age, for example, then that positive feedback probably helped me get over that’* (P2); *I tried it one more time, just local, but yes, by then I think friends were saying to me they thought it was too risky and I shouldn't do it’* (P4); ‘*The instructor was very good. The second lesson where we went for maybe a 45-min ride on the road and the cycle paths, that was good, and they’re always there for help’* (P12)
5. Reflective motivation	g. Optimism (15)	**i. Optimistic that e-cycling would positively impact health** (15) (F)	*‘I'm hoping cycling will help* [manage diabetes]*. I've always tried to do a bit of exercise. But I've also cut back on one of my medication as a result of health problems, so I'm hoping that this is going to equate to the extra Metformin’* (P1); ‘*It was all health [the reason for signing up to the study]. I wasn't really thinking about the environmental factor, at all. I just thought it was something I needed to do’* (P16); ‘*But [Instructor 1] was very good. The second lesson where we went for maybe a 45-min ride on the road and the cycle paths, that was good, and they're always there for help. If you did have a problem, they're always at the end of the phone’* (P12)
	h. Beliefs about consequences (122)	**i. Ability to ride further, longer, hillier terrain and new routes** (31) (F)	*‘I mean, even the steepest hills were easy enough to go up on the e-bike, you just turn down the gears and put on the turbo’* (P1);*‘In the knowledge that I had the assistance, I would tackle routes and push myself a bit further than I would normally on my own cycle’* (P16)
		**ii. Environmental and financial impact of e-cycling** (31) (F)	*It saves money because I don't have to find bus fare. So, it will have a financial impact when I buy one, but after that I think I would definitely save money.* (P7)
		**iii. Long- and short-term health benefits of e-cycling** (60) (F)	*‘Yes, my legs are definitely stronger than what they used to be. I've got more energy to do things’* (P7); ‘*I had more stamina through the day riding it. Yes, I felt better. More alive. Usually I just sit, like I'm doing now, on the couch, with the cat and the television on’* (P3)
	i. Beliefs about capabilities (135)	**i. Confidence riding on roads or in traffic** (65) (B, F)	*‘I quite happily went out with the traffic. Traffic doesn't bother me that much, as much as I am a driver and I do cycle, so it wasn't much of a problem. I felt confident enough amongst it’* (P16, lots of cycling experience); *I think it decreased [confidence]. Because I think I was a bit taken aback because I had expected to not be nervous, because I’ve cycled before. So, it was a bit of a surprise. When I headed off to do my journey, I was completely confident, you know, that it wouldn't be difficult. So, it was a bit of a surprise, yes’* (P4, lots of cycling experience).
		**ii. Perceived competence riding an e-bike** (70) (B, F)	*I have to be honest, I never felt comfortable with the bike that I was given because I found the frame too high’* (P2, lots of cycling experience); *When I started, I found I was more uncomfortable on a bike than I thought. I did find the e-bike slightly heavy to handle so I was a little bit uncomfortable in traffic to start off with. But my confidence grew the more I went out and I think now I'm fairly confident on the bike and it's certainly handling better* (P7, lots of cycling experience); ‘*As far as riding it is concerned, a little bit lacking in confidence to begin with, because it's quite big and quite heavy and you're quite high up, but over time, I gained my confidence with it and worked out how to use the gears and engine quite efficiently’* (P11, a little cycling expeirence)
	j. Social professional role and identity (29)	**i. Perception of self as a cyclist** (17) (F)	*‘My views before, because I am not a cyclist, or I consider myself I am now but before I wasn't a cyclist and I thought I never would. But the trial coming along just gave me that confidence and just changed my total outlook on it, but I do feel I am a cyclist now’* (P12); ‘*I regard myself more as a cyclist than I did before and if it wasn't for this project I wouldn't have even considered getting out on the bike’* (P7)
		**ii. Cycling advocacy** (12) (F)	*‘I've spoken to quite a few people about them and said, “You've got to give them a go, they're loads of fun and if you don't like cycling, it'll make it a lot easier for you to go back onto a bike”. As I said, we've even got one for use of people at work and I keep saying to various people, “Go on, jump on it and have a go, you'll have some fun."’*(P11)
	k. Goal (11)	**i. Planning and scheduling e-cycling** (11) (F)	*I remember in the diary, when I was talking with [the instructor] and I put all my rides in for a 12-week period and then there were targets and I said I'd like to ride to work just once in the trial, if I can get to work.* (P12); *But what I do think I did do was try to achieve targets, so try to achieve, “I'm going to go and see if I can make it to Bath and back and increase the distance. So, I would say I used it less regularly than I thought, but I achieved better targets than I thought I'd be able to do* (P11)
	l. Intentions (81)	**i. Desire to continue riding** (40) (F)	*‘I've applied for the cycle scheme and I'm just waiting for the voucher to come through; I've already decided what bike to get, it's a Raleigh Motos Grand Tour. It can do as much as 150 miles on one battery’* (P12); ‘*Well, again, it's now work hard, save up the money, and get one’* (P13)
		**ii. E-cycling intentions (**41) (F)	*My intentions were to take it to work. Then when you hear of people having their bikes stolen and I'm thinking, “Hang on a minute’* (P8); *‘**At the start of the programme, how did you think you were going to use the bike? ‘**Just leisure. I planned to commute once. Then I started going longer and I thought it's a little bit too far to cycle to work and I thought I'd just do it once and then I must have done it maybe a dozen times or maybe twenty times during the trial. I find it a lot easier than I thought it was going to be’* (P12)
6. Automatic motivation	m. Emotion (35)	**i. Riding the e-bike enjoyable and satisfying** (35) (F)	*‘There were routes that I chose with the electric bike that I haven't embarked on with my ordinary cycle, despite having it 27 years…. I must admit, I found it quite a rewarding experience’* (P16); ‘*It's actually a pleasurable experience, riding an e-bike, to be honest, it takes out the grind of going up and down hills, so it takes out the… it makes the difficult parts of cycling become more enjoyable’* (P1); ‘*I thought it was such a nice morning, I came back through the country route, so I came back over the common. That's how much I enjoyed it. I extended the distance and the time it took me to return home’* (P7); ‘*I sort of had to map it out before I went but actually when I started off, I actually really enjoyed it. I went down the river for a bit and then I went up into XX and it was lovely, really exhilarating’* (P14)
	n. Reinforcement (25)	**i. Habituation of e-cycling** (17) (F)	*Certainly, in terms of taking it to work, yes. It was a case of it was a no-brainer. There would be no reason that I wouldn't take the bike.* (P11): ‘*No, I wouldn't say I got into the habit of riding it on a daily basis. I wish I did, but I just rode it whenever I could. But certainly, I think it's a habit I could get into’* (P7)
		**ii. Sense of achievement** (8) (F)	*It makes you feel yourself, makes you feel better in yourself when you actually achieve what you set out to do* (P1)

The bold text here denotes the interviewee speaking.

Before starting the e-bike training participants with previous cycling experience felt that the training was going to be unnecessary. However, all participants reported learning new skills, specifically how to handle the e-bike and use the electrical assistance, and how to ride safely on roads and in traffic (Table 3.1.a.i/ii). The training conducting during the loan period enabled participants to practice their skills.

##### Psychological capability

###### Knowledge

The majority of participants were aware of the benefits of engaging in PA, in general, and for diabetes management (Table 3.2.b.i). Several participants felt that engaging in PA was an easier way of managing their diabetes than dieting. This knowledge impacted their desire to sign up for the trial and engage in e-cycling.

The e-bike training provided participants with sufficient knowledge on how to use the e-bike. Over time, with practice, participants became more efficient at using the gears and electrical assistance (Table 3.2.b.iv). In addition, the training taught participants how to safely ride on the road and where to find safe cycle paths (Table 3.2.b.ii/iii). When choosing where to ride some participants reported sticking to familiar routes, for which they knew the cycling infrastructure and/or traffic levels. While others enjoyed using the cycling maps to plan rides and explore new bike paths (Table 3.2.b.ii).

###### Behavioural regulation

Although infrequently discussed, some participants reported that monitoring their behaviour using tools such as Strava or a GPS watch encouraged them to engage in e-cycling.

###### Memory, attention and decision making processes

Participants reported evaluating the pros and cons of cycling vs. using a different mode of transport (Table 3.2.d.ii). As such, the decision to engage in e-cycling was often carefully considered. In general participants felt that utilitarian journeys required more organisation than recreational journeys. However, participants who had cycled regularly in the past reported having strategies to ensure they would remember all the required equipment, such as prepacking bags or running through mental checklists before leaving to ensure they had all their equipment (Table 3.2.d.i).

#### 3.2.2. Opportunity

##### Physical opportunity: *environmental context and resources*

Barriers relating to the physical opportunity were the most commonly reported by participants in this study and included:.

###### Cycling infrastructure, parking facilities and traffic

Participants were concerned that the e-bike could be stolen if left in a public space, especially when the parking facilities were deemed inadequate (Table 3.3.e.viii). Theft anxiety was exacerbated as the e-bike was on loan and participants were unclear of the financial implications for themselves if the e-bike was stolen. Participants reported that they would have been less anxious about locking the e-bike up in public spaces if it belonged to them. Having good parking facilities at a destination facilitated e-cycling, particularly for utilitarian purposes, while poor parking facilities inhibited e-bike use (Table 3.3.e.i). Having limited e-bike storage at home also inhibited e-cycling. Several participants had to keep the e-bike indoors which meant getting in and out the house required considerable effort (Table 3.3.e.i).

Regarding riding, participants were reluctant to ride on roads with no cycling infrastructure, primarily due to traffic concerns (Table 3.3.e.vii). Participants who felt they were close to segregated cycle paths were willing to cycle short distances on the road to reach these paths. Participants’ level of confidence riding the e-bike and riding on roads impacted the degree to which segregated cycling infrastructure was deemed a necessity. For those with limited confidence riding on roads, not having easy access to segregated paths negatively impacted their e-cycling. For participants with high levels of confidence riding on the road the absence of cycling infrastructure, while not enjoyable, did not stop them from engaging in that specific ride. There was concern regarding the volume of traffic on cycle paths and the complications of mixing cyclists and pedestrians due to travelling at variable speeds. In addition, a few participants reported that in the dark the cycle paths felt isolated, and they felt vulnerable (Table 3.3.e.i).

###### Time and weather constraints

Work and personal caring responsibilities inhibited e-cycling for some participants, particularly those in full-time employment who felt they had limited time (Table 3.3.e.vi). Heavy rain and darkness were the two weather related barriers frequently reported by participants (Table 3.3.e.iv). For some participants having wet weather gear and good lights helped to overcome these issues, while others actively chose not to cycle in these conditions despite access to equipment.

###### E-bike specific issues

The weight of the e-bike was noticeable to all participants and in some cases made the e-bike hard to manoeuvre (Table 3.3.e.ii). In addition, for some the e-bike frame was perceived as being too large, leading to feelings of discomfort. Many participants, potentially due to the weight of the bike, wanted to be able to comfortably put their feet on the ground when stationary. Issues with the perceived size and weight of the frame impacted participants confidence both riding the e-bike and riding on the road. In addition, participants found the battery itself to be inconvenient and heavy to carry around.

Several participants commented that they would like to continue cycling after the trial but that the cost of the bike meant they were unable to do so. For some participants this meant changing to conventional cycling, while for others this meant not engaging in cycling until they had saved sufficient money to purchase an e-bike (Table 3.3.e.iii).

###### Personal health issues

During the study personal health issues impacted cycling for several participants. These were both acute and chronic in nature. The chronic conditions occurred in participants over 60-years of age, while acute conditions occurred in younger participants. Four participants reported having pre-existing additional health conditions. For two participants this negatively impacted riding (arthritis in hand and difficulty lifting their arm to signal due to cancer treatment) while the other two were able to develop strategies to deal with the health condition (blind in one eye and hearing loss in one ear) (Table 3.3.g.v).

##### Social opportunity

When instructors were perceived as engaged in the training, participants reported feeling practically and emotionally supported. These instructors delivered more training sessions and participants felt that they practiced riding in areas that they may not have previously considered. However, instructors perceived as being disengaged conducted less training sessions and participants did not feel supported through the training (Table 3.4.f.i/ii).

Outside of the e-bike training, the amount of practical support received and/or desired ranged greatly between participants. Participants with no previous cycling experience desired more practical support to enable or motivate them to ride than those who knew how to cycle (Table 3.4.f.i). Several participants reported cycling with friends and family which was enjoyable and motivational (Table 3.4.f.i). There appeared to be a gender difference with men being content to cycle alone and women desiring greater practical riding support.

Similarly, some participants found verbal support to be encouraging, while others felt their decision to ride was not influenced by others. For individuals who were unsure about whether e-cycling was appropriate for them the feedback from others was impactful, either encouraging them to continue e-cycling or confirming their decision to stop. For one participant, who was struggling with e-cycling, the feedback from their friends impacted their decision to stop riding (Table 3.4.f.ii). There were no apparent differences in the perception of verbal support based on gender.

#### 3.2.3. Motivation

##### Reflective motivation

###### Optimism

Participants primary motivation for signing up for the study and engaging in e-cycling was to have a positive impact on their health rather than due to environmental concerns (Table 3.5.g.i). Given their knowledge of the benefits of PA, participants reported being optimistic that e-cycling would positively impact their health (Table 3.5.g.i).

###### Belief about capabilities

For the majority of participants the e-bike training positively impacted their perceived competence to ride the e-bike and over time, with increased practice, this confidence grew (Table 3.5.g.ii). Participants with recent cycling experience (two to three years) were more confident riding an e-bike on roads and in traffic compared to those that hadn't ridden for a while (Table 3.5.g.i). However, for some participants, even previous cyclists, the unanticipated differences between conventional cycling and e-cycling, as well as the poor conditions of the road negatively impacted their riding confidence (Table 3.5.g.i).

The weight and size of the e-bike negatively impacted confidence riding the e-bike for some participants (Table 3.5.g.ii). Participants reported how the instructors made a series of alterations to the seat height or provided a smaller size e-bike to increase their comfort and ability to ride. With practice, and alterations to the equipment, participants’ felt they were able to ride the bike and became more confident (Table 3.5.g.i/ii).

Overall participants’ degree of confidence riding the e-bike impacted their confidence riding on the road. Specifically, participants who were more confident riding the e-bike were also more confident riding on the road with traffic. While those who were uncomfortable on the e-bike reported greater anxiety riding on the roads due to not being able to respond to changing situations and interactions with cars.

###### Belief about consequences

Beliefs about the consequences of engaging in e-cycling were the most commonly reported facilitators to engagement. Specifically, both men and women felt that e-cycling positively impacted a variety of health outcomes including a) diabetes management, through notable decreases in blood sugar levels, b) improved mental health and c) increased fitness (Table 3.5.i.iii). By comparison, few participants felt that e-cycling had significant financial or environmental implications for themselves.

One of the most commonly reported facilitators to e-cycling was the electrical assistance which enabled participants to travel further, faster and on hillier terrain in comparison to a conventional bicycle (Table 3.5.i.i). This contributed to e-cycling engagement and enabled participants to try out routes that they would not have considered tackling on a conventional bicycle.

###### Social identity

Using an e-bike regularly for three months enabled some participants to feel more like a cyclist. However, the degree to which participants identified as a cyclist varied greatly. Specifically, participants who reported a stronger e-cycling habit, and greater distances travelled, saw themselves more as cyclists than those who did not get in the habit of cycling regularly and travelled less distance (Table 3.5.j.i). Three participants shared how they would advocate e-cycling to others, while another three reported having specific discussions around the benefits of e-cycling and saw themselves as role models for e-cycling (Table 3.5.j.ii).

###### Goals

Participants who rode a greater distance over the trial period discussed setting a range of e-cycling goals including riding to a certain location, using less assistance or completing a route in a faster time (Table 3.5.k.i).

###### Intentions

Prior to the study participants planned to use the e-bike for both recreational and utilitarian purposes. However, for those that intended to make utilitarian journeys via e-bike, and replace other transport modes, they discussed how they were unable to achieve this intention due to a range of environmental barriers identified above (Table 3.5.l.ii). Though one participant, who planned to use the e-bike purely for leisure, used it primarily for commuting (Table 3.5.l.ii). This individual felt able to commute on the e-bike due to access to safe parking infrastructure at their place of work.

At the end of the study, 12 participants wanted to continue riding an e-bike, with five actively seeking out e-bike purchasing options and one purchasing an e-bike part way through the study (Table 3.5.l.i). Two participants wanted to continue riding on a conventional bicycle following the study. Of those seeking out e-bikes half reported that an e-bike was out of their price range and expressed a need to save up or wait for a change of circumstances (e.g., retirement).

##### Automatic motivation

###### Emotion

The enjoyment associated with e-cycling was a key facilitator to engagement. Specifically, the ability to ride new and longer routes meant e-cycling led to feelings of enjoyment (Table 3.6.m.i). Part of the enjoyment of e-cycling came from being in the fresh air and nature and having the ability to explore new routes. Cyclically, the enjoyment associated with riding led participants to ride further and more frequently than they had anticipated.

###### Reinforcement

Participants reported experiencing a sense of achievement once a ride was complete (Table 3.6.n.ii). This achievement, along with the enjoyment, led to increased riding and some participants commented how this helped e-cycling to become a habit for them (Table 3.6.n.i).

## 4. Discussion

This theory-based qualitative study examined the factors associated with engagement in e-cycling among individuals with type 2 diabetes, with a particular focus on identifying barriers and facilitators to engagement. In addition, the study sought to develop a conceptual understanding of how these factors relate to one another to impact behaviour. This information can be used to refine the current intervention (57) and/or guide future initiatives aimed at increasing e-cycling in people living with chronic disease. The key findings of this study are discussed below.

### 4.1. The importance of e-bike training to build actual and perceived capability

The results of this study highlight that regardless of previous cycling experience e-bike training is beneficial, providing participants with riding skills, knowledge of how to safely ride on roads and where and how to access segregated cycling infrastructure. Consequently, participants reported increased confidence when riding the e-bike, riding on the road and exploring new cycling routes. The increase in confidence was greatest among individuals who had not cycled for a considerable period of time. Similar increases in confidence have been reported among older adults in the UK following e-bike training (58). Furthermore, a recent review of conventional bike skills training programmes concluded that training led to increased riding confidence, and was positively associated with increased cycling frequency (29). These findings highlight the importance of training to target key individual level predictors of e-cycling, namely skills, knowledge, and confidence.

Despite the apparent positive impact of training on cycling behaviour, e-bike interventions rarely report the details of training provided. This maybe because no formal training is conducted, training is minimal, or researchers do not consider the impact of the e-bike training on behaviour. Lack of reporting of intervention content and duration is a similar problem in conventional cycling studies (29). An understanding of what is delivered as part of an e-cycling intervention is important to determine what is most likely to facilitate e-cycling. E-bike training is of particular importance to older adults or people living with chronic disease who may have pre-existing health concerns that require adaptations to the e-bike, riding style or riding location. These issues can be addressed and overcome with support from an instructor, as was the case in the current trial. It is important to note that in the current trial three participants, (two with a little cycling experience and one with no experience) required more than the specified four training sessions, highlighting the need to tailor e-bike training.

In addition, more support was desired for women who had less cycling experience than men when entering the study, a finding echoed in other e-bike trials (59). In the current trial men completed higher levels of skills training (National Cycling Skills levels two and three) than women prior to taking the e-bike home and cycled further, on average, than women during the trial. Previous cycling research has shown that higher levels of national skills cycle training completed is associated with more riding (60). As such, women should be supported to reach these higher levels of skills training prior to an e-bike loan.

Instructor led cycling sessions conducted during the e-bike loan made participants feel supported and offered an informal setting in which to discuss e-cycling and practice riding. Furthermore, participants that attended these sessions rode further than those that did not. Serali and colleagues (29) found that cycling frequency decreased over time following training and recommend that training should be followed up by post training support to ensure that participants consolidate the skills and confidence gained during training. This recommendation is supported by the findings of the current study and suggest that the additional support provided in the current study, above and beyond delivering skills training, is important to practice skills and maintain confidence. Conversely, when participants reported instructors to be disengaged in the training this negatively impacted confidence. As such, instructors need to be comprehensively trained, not only on the skills component, for which they were confident (49), but also on how to offer support and effectively engage with a population who may require more support than the instructors are used to providing.

### 4.2. E-bike size and weight concerns

Despite comprehensive training several participants reported that the e-bike was too large making it difficult to manoeuvre, leading to decreased confidence riding the e-bike in general and in traffic. While the e-bikes provided were an appropriate size based on participants’ height, participants wanted to be able to fully place their feet on the ground. E-bike size concerns are not a commonly reported barrier to e-cycling and could be due to the characteristics of this sample. Specifically, the current sample were classified as obese and had extremely low fitness levels which could have negatively impacted balance (49). In addition, a type 2 diabetes diagnosis is associated with reduced balance (61–63). This reduced balance and low fitness could have meant participants found it hard to manage the weight of the e-bike when stopping and starting. In the current study the provision of a smaller frame size, which enabled the participant to fully plant their feet on the ground, was associated with increased confidence riding the e-bike and riding on roads. As such, the provision of smaller e-bike frames than is standard would likely increase riding confidence and engagement in e-cycling in this population.

### 4.3. Motivational factors that impact e-cycling

Participants were motivated to engage in e-cycling to improve their health as opposed to impacting the environment. This optimism regarding health was largely met, with participants perceiving improvements in fitness, mental health and diabetes management, findings echoed in previous e-cycling research (42). Engaging in e-cycling was perceived as an easier way of managing their diabetes than diet or other types of exercise, largely due to the enjoyment of riding. Enjoyment came from the ability to ride a bike comfortably due to less physical exertion than a conventional bicycle and the ability to ride further, faster and on hillier terrain than previously possible. These benefits are consistently reported in the e-cycling literature (34). A substantive body of literature now demonstrates that positive enjoyment during exercise is associated with greater future engagement (64) and is a unique aspect of e-cycling over other forms of active travel. High levels of enjoyment appeared to increase the habit of e-cycling in the current sample, with participants who felt e-cycling had become habit accumulating greater kilometres ridden than those who did not.

### 4.4. The need for social support

In the current sample, the degree to which support was required, or desired, varied based on level of experience and gender. Specifically, individuals with low levels of cycling experience, who were primarily women, required and desired more practical support from both the instructor and friends and family. One participant attributed their inability to become an independent cyclist on a lack of social support in their personal life. In addition, women reported wanting to ride with friends or family to a greater extent than men. Conversely, men reported that e-cycling alone was relaxing and enjoyable. This has been reported in other e-cycling studies among older adults (58, 65). In the current study verbal support was less influential than practical support. This may be due to the higher-than-average rates of cycling in Bristol and potentially a community acceptance of cycling in general (66).

### 4.5. The impact of the natural and built environment

Access to safe parking infrastructure was a commonly reported barrier to utility e-cycling. Specifically, a lack of safe parking facilities and fear of theft negatively impacted riding. While these are commonly reported barriers to cycling (34, 67) these fears were exacerbated in the current study due to the e-bike not being owned by the participant and concerns over the financial implications of e-bike theft. While there is scant evidence of the impact of bicycle parking in cities on cycling behaviour, Heinen and colleagues report that the supply and quality of parking can impact cycling behaviour (68).

Home parking facilities were also a concern for some participants. Specifically, participants reported having to park the e-bike in the house and the effort required to get the bike in and out negatively impacted riding. Very little research has explored the impact of home parking facilities on cycling behaviour. This concern maybe more pertinent to e-bikes which are heavier and bulkier than conventional bicycles (69). In the current study two participants reported regularly commuting to work. These individuals reported having access to safe bicycle storage and showers and in one case the company had restricted car parking making e-cycling more attractive. Workplace facilities and polices such as these have been found to be positively associated with cycle commuting (70–73).

The cycling infrastructure to which an individual had access also impact riding. Specifically, participants were more willing to cycle when they had access to a segregated cycling path close to their home. Providing infrastructure that supports the needs of cyclists is recognised as a key strategy to encourage more cycling in cities (74–76). Two recent systematic reviews show that cycling behaviour increased following the introduction of new infrastructure or upgrading existing infrastructure (77, 78), however evaluation of environmental interventions is complex and findings vary based on the method of evaluation used (79).

Overall factors associated with the natural and built environment were instrumental in participants decision to engage in e-cycling or take an alternative mode of transport. In some cases, participants removed any notion of utility cycling and stuck to recreational rides due to these barriers, this was particularly relevant for individuals with no or little previous riding experience. While participants were encouraged to engage in problem solving and action planning to overcome such barriers, the extent to which individuals engaged in these activities at appropriate times is unknown.

### 4.6. The financial cost of e-bikes

Trialling an e-bike led to 12 of the 16 participants wanting to purchase an e-bike to continue riding, largely due to the high level of enjoyment. This is in line with other research which has reported that the desire to purchase an e-bike substantially increases following a e-bike trial and is associated with enjoyment, positive attitudes towards e-bikes and perceived benefits (59, 80). While many individuals are willing to pay the large expense of an e-bike (59, 80, 81) others, including individuals in this study, although willing, do not have the financial security to be able to purchase an e-bike (59). Following completion of a trial period, purchasing an e-bike has been reported to be an independent predictor of e-bike use over time (59) and is associated with reducing an individual's habitual use of the car (82). As such, ways of helping participants to view e-bikes as a financially viable option is of upmost importance.

### 4.7. Strengths and limitations

A strength of this research is the use of the TDF and COM-B to examine experiences of e-cycling in this population. To the authors’ knowledge this is the first time this framework and model have been used to explore peoples understanding and perception of e-cycling. Their use allow for the exploration of factors beyond skills and knowledge-based considerations and for the development of an understanding of the impact of context on e-cycling engagement. This information can be used to develop a programme theory, identifying hypothesised causal pathways, which can be tested in future trials. However, there are several limitations. Firstly, using the thematic approach means that data are combined and summarised, so that individual level detail maybe lost (83). In the current analysis the aim was to bring forward unique cases into the matrix and report these in the results. Secondly, thematic analysis focuses primarily on what the data show, thus failing to consider potential areas that are not discussed. While this is hard to avoid, use of the TDF ensured that a wide range of topics were covered. Thirdly, telephone interviews have been suggested to be inferior to face-to-face interviews due to a lack of visual cues, however, there is limited evidence to support this statement (84). Rather, telephone interviews may enable participants to share information more openly than face-to-face interviews, and participants maybe more relaxed when talking from the comfort of their own home (84). Fourthly, the role of the researcher may have impacted the data obtained, the analysis and the findings. The prior relationship that the researcher, who conducted the pilot RCT, had with participants may have impacted how participants responded to questions. To try and overcome this a distinction was made between the e-bike training and the study, to help the participant view them as different components to encourage honest opinions to be shared. Participants were asked to give frank answers to enable intervention improvements. In addition, the researchers who conducted the analysis were commuting cyclists at the time of the study. This may have influenced interpretation of the data. However, interpretations made by the researchers and reviewed by the participants showed consistency, therefore increasing the trustworthiness of the findings. Fifthly, 94% of the sample identified as White. As such, these findings cannot be generalised to other ethnicities. However, a strength of the study is the equal inclusion of men and women. Sixthly, 14 of the 16 participants had some degree of cycling experience, which may bias the identified barriers and facilitators to e-cycling reported and therefore the current findings cannot be generalised to individuals new to cycling. Individuals who had recent cycling experience (i.e., in the last two or three years) had greater perceived capability and confidence than those with little or no cycling experience. While one individual who had never previously cycled was unable to e-cycle following instruction. However, the degree to which previous cycling experience impacted current e-cycling behaviour depended largely on the individual and additional contextual factors.

### 4.8. Implications for future research

Using the information obtained from this analysis, researchers should refine the intervention to address some of the highlighted concerns using the Behaviour Change Wheel. Following refinement, a programme theory should be developed to generate hypotheses about how the intervention impacts cycling in this population. This can be used to guide the selection of quantitative mechanistic outcomes and contextual variables that should be examined in a future trial. Further research should be conducted to differentially examine the impact of such e-cycling interventions on individuals new to cycling or who have not ridden for a considerable amount of time, as the degree of training required appears to be different to those who are confident with cycling. In addition, such an intervention should be trialled in different populations to allow comparison of experiences.

While training is important, it needs to be part of a multifaceted approach including improving infrastructure and introducing policy to encourage e-cycling engagement. Future research should involve working with stakeholders to establish how to address some of the contextual barriers to e-cycling, specifically cycling and parking infrastructure and traffic concerns, and the impact that addressing these components has on e-cycling engagement. In addition, means through which to address the financial cost of initiating e-cycling should be explored. Examining the relative impact of these micro-environmental, social and individual factors, and their associated costs, will provide guidance on how best to promote e-cycling in the future and highlight the potential for scalability.

## 5. Conclusion

Findings from this study provide insight into the personal, social and environmental factors that individuals with type 2 diabetes report as barriers and facilitators to e-cycling. Using the TDF and COM-B model is a starting point to understanding e-cycling in the current context and identifying what needs to change to modify the behaviour. This can inform the development of a conceptual framework which hypothesizes how these factors impact one another to influence e-cycling behaviour. This is the first step in developing an understanding of the mechanisms through which the e-bike training impacts individual capability and motivation, and identifying the importance of different environmental and social factors on e-cycling engagement. The findings of this study can be used to improve the quality of bicycle skills training currently being offered and to guide the selection of mechanistic outcome measures for future evaluation, guide scale-up to other locations and inform policymakers of what further actions need to be taken to enable people to adopt bicycling.

## Data Availability

The raw data supporting the conclusions of this article will be made available by the authors, without undue reservation.
