# Supplementary material for: ‘‘I felt marvellous e-cycling. If I had long hair I would have flicked it”: a qualitative investigation of the factors associated with e-cycling engagement among adults with type 2 diabetes
Source: Front Sports Act Living. 2023 Sep 29;5:1150724. doi: 10.3389/fspor.2023.1150724 (PMC10570523; doi:10.3389/fspor.2023.1150724)
Supplement: Supplementary file 1 [file Datasheet1.pdf]

Supplementary Material: Interview guide

Rationale for Question	Interview Questions
**Background – all participants**	
Background<br>*To find out where participants are located in the city and their work situation as this could impact perception of e-cycling or the study procedures*	Where do you live?<br><br>Are you working? (Regular 9-5 or shift work?)
**Process evaluation questions – all participants**	
Broad questions to start discussion<br>*Asked to reflect on study*	Tell me about your experience of participating in the study?<br><br>Which of the assessments do you remember?<br><br>Which things did you enjoy/not enjoy about taking part?<br><br>What did you think about taking part in the different assessments?<br><br>How could we have improved any of the assessments or the study in general?
**Theoretical Domains Framework**	
Knowledge:	Tell me how you felt riding the e-bike? (to being with and also at the end)<br><br>How did you get on with the e-bike to being with and at the end (PROMPT: comfort, handling, technical aspects, use of assistance, utility etc.)<br><br>Was it as you expected? (PROMPT: In what way)
Skills:	Have you done much cycling in the past (PROMPT: As a child, adult)<br><br>Do you feel as though you were provided with adequate training on how to ride an electric bike?<br><br>Would you have liked more training before taking the e-bike home?
Social/Professional Role and Identity:	Has using the e-bike made a difference to the way you see yourself in relation to being a 'cyclist'?<br><br>Did you purchase/acquire any equipment for cycling? (e.g., clothes, lights, gloves etc.) during the intervention or do you plan to?
Beliefs about Capabilities:	Did you feel confident riding the electric bike? (At the start, did this change over time?)<br><br>Were there specific situations in which you felt more or less confident?

	Prompt: different kinds of roads, heavy traffic, riding with others?<br><br>What would help you feel more confident/make it easier to ride the bike?
Optimism:	At the outset, did you feel that the intervention would be positive for other things, e.g. how you feel, how much activity you do?
Beliefs about Consequences:	Tell me about e-biking in relation to your health?<br><br>Do you think e-cycling has the potential to help you manage your diabetes? If so, in what way can if help?<br><br>How does e-cycling compare with other self-management behaviours (medicine, diet, other physical activity?)<br><br>What do you think about the impact of e-biking on the environment? (e.g., replace car journeys, bus etc)<br><br>Does choosing to e-bike have a financial impact for yourself?<br><br>Do you think there are any negative outcomes or harms associated with e-biking?
Reinforcement:	Would you say you are in the habit of riding you bike on a regular basis?<br><br>If not – what would be helpful in developing more of a routine/habit for biking?
Intentions:	At the start of the program, how did you think you would use the e-bike? (e.g., commuting, leisure, shopping, social)<br><br>Did these intentions match how you used the bike? (expand)
Goals:	To what extent was riding the bike a priority for you during the intervention?
Memory, Attention and Decision Processes:	Can you tell me about how you made decisions about using the bike on a day to day basis?<br>- PROMPT – feelings, environment, weather, sweaty, effort?<br><br>How did location and infrastructure (roads, cycle routes/paths, terrain etc) influence your decision to e-cycle?<br><br>How easy or difficult was it to remember all the accessories you needed when riding the bike (e.g., panniers, lights, clothes)?<br><br>Can you give me an example of the types of journey you made on the e-bike? Can you tell me why you decided to use the e-bike for this journey?

Environmental Context and Resources:	What barriers did you experience riding the e-bike on a day-to-day basis? (PROMPT: equipment, weather, time or competing interests, pressure from others)<br><br>Did you have all the equipment to ride the e-bike?
Social Influences:	Tell me about the training you received on how to ride an e-bike? How did it make you feel?<br><br>How did you feel about the follow-up sessions with the instructors?<br><br>Did your friends and family express any opinions about you riding the e-bike?<br><br>Did their view make any difference to your bike riding?<br><br>Did you join any organized cycling rides or ride with others (expand)?<br><br>Do those closest to you cycle (Friends or family)?
Emotions:	When we ask about riding the e-bike what emotions come to mind?
Behavioural Regulation:	Did you have any systems in place to ensure that you rode the e-bike on a regular basis? E.g. reminders, set rides with other people, particular places?
Closing	
	What is your perception of cycling after participating in this study?<br><br>How do you feel about coming to the end of the study and handing the bike you loaned back?<br><br>Do you have any plans or are you interested in looking at options to carry on e-biking?<br><br>What would you say to other about e-bikes and being in the study?
